# Supplementary material for: Asynchronous Rate Chaos in Spiking Neuronal Circuits
Source: PLoS Comput Biol. 2015 Jul 31;11(7):e1004266. doi: 10.1371/journal.pcbi.1004266 (PMC4521798; doi:10.1371/journal.pcbi.1004266)
Supplement: S1 Text — (PDF) [file pcbi.1004266.s001.pdf]

Supporting Information for the article
"Asynchronous Rate Chaos in Spiking Neuronal Circuits"

Omri Harish and David Hansel

S1 Finite size effects in inhibitory rate networks with sigmoid or threshold-linear transfer functions

Deviations between simulations and dynamical mean field theory (DMFT) are expected since the latter assumes networks of infinite size. To investigate whether the deviations we observed between our simulation results and DMFT predictions were due to such effects we simulated networks of increasing size $N = 32{,}000$, 64,000, 128,000 and 256,000. Averaging the results over 8 realizations of the networks for each size provided estimates, $\sigma_0^{sim}, \sigma_\infty^{sim}$, μ^{sim} of the quantities, σ_0, σ_∞ and μ (see *Materials and Methods*). We compared these estimates to the corresponding DMFT predictions, $\sigma_0^{th}, \sigma_\infty^{th}$ and μ^{th}. Figure S1 plots the dependence of the deviation between the theoretical and simulated values on network size, for inhibitory rate models with sigmoid (Fig. S1A) or with threshold-linear (Fig. S1B) transfer functions. The results indicate that the differences between DMFT predictions and simulations become very small for infinite N and that this happens faster for σ_∞ than for σ_0 and μ. Note, however, that small discrepancies should persist even in the limit of infinite N. This is because the DMFT assumes Gaussian statistics of the net inputs into the neurons, an assumption which becomes exact only when the connectivity, K, is infinite. However, for the values of K used in the simulations displayed in Fig. S1 the finite K corrections were much smaller than the finite N corrections (results not shown).

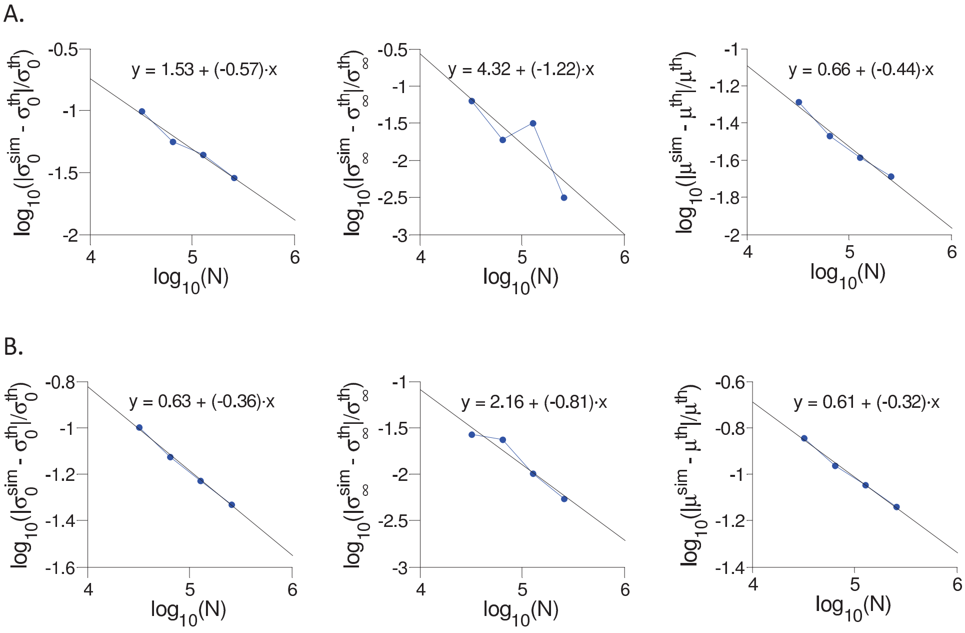

Figure S1: **Finite size effects in inhibitory rate models with sigmoid or threshold-linear transfer functions.** The relative difference between the simulation results, $(\sigma_0^{sim}, \sigma_\infty^{sim}, \mu^{sim})$, and the predictions of the DMFT, $(\sigma_0^{th}, \sigma_\infty^{th}, \mu^{th})$, are plotted vs. the network size N (in log scale) for σ_0 (left), σ_∞ (middle) and μ (right). A: Sigmoid transfer function, $g(x) = \frac{1}{2}\left[1 + \mathrm{erf}\left(x/\sqrt{2}\right)\right]$, $I_0 = 1$, $J_0 = 15$, $K = 2000$. B: Threshold-linear transfer function, $g(x) = \max(x, 0)$, $I_0 = 1$, $J_0 = 2$, $K = 1200$.
