# Supplementary material for: Asynchronous Rate Chaos in Spiking Neuronal Circuits
Source: PLoS Comput Biol. 2015 Jul 31;11(7):e1004266. doi: 10.1371/journal.pcbi.1004266 (PMC4521798; doi:10.1371/journal.pcbi.1004266)
Supplement: S2 Text — (PDF) [file pcbi.1004266.s002.pdf]

Supporting Information for the article
"Asynchronous Rate Chaos in Spiking Neuronal Circuits"

Omri Harish and David Hansel

S2 Chaos onset in inhibitory rate models with twice differentiable transfer functions

Numerical simulations indicate that for all the transfer functions considered in this paper the bifurcation from fixed point to chaos is supercritical. When $J_0 \to J_c^+$, σ_0 and $\sigma_\infty = \sigma_{max}$ as well as σ_{min} converge to σ_c. The first and second order derivatives of $V(\sigma;\sigma_0)$ with respect to σ must therefore be equal to zero at σ_c. Thus σ_c, J_c and the value of at the bifurcation, μ_c, are determined by:

$$\sigma_c = {J_c}^2 \int_{-\infty}^{\infty} \left[g\left(\mu_c + \sqrt{\sigma_c} z\right)\right]^2 \mathrm{D}z \tag{1}$$

$$1 = {J_c}^2 \int_{-\infty}^{\infty} \left[g'\left(\mu_c + \sqrt{\sigma_c} z\right)\right]^2 \mathrm{D}z \tag{2}$$

$$I_0 - \frac{\mu_c}{\sqrt{K}} = J_c \int_{-\infty}^{\infty} g\left(\mu_c + \sqrt{\sigma_c} z\right) \mathrm{D}z \tag{3}$$

.

To study the critical behavior at chaos onset we expand the DMFT equations in $\delta = J - J_c \ll 1$. Defining:

$$\mu = \mu_c + \mu^{(1)}\delta + O(\delta^2) \tag{4}$$

$$\sigma_0 = \sigma_c + \sigma_0^{(1)}\delta + O(\delta^2) \tag{5}$$

$$\sigma_\infty = \sigma_c + \sigma_\infty^{(1)}\delta + O(\delta^2) \tag{6}$$

and assuming $g(x)$ continuously differentiable to the second order, we expand $V(\sigma)$ around σ_∞:

$$V(\sigma) = V(\sigma_\infty) + \frac{1}{2}V''(\sigma_\infty)(\sigma - \sigma_\infty)^2 + \frac{1}{6}V'''(\sigma_\infty)(\sigma - \sigma_\infty)^3 + ... \tag{7}$$

where we used $V'(\sigma_\infty) = 0$. Since $V''(\sigma_c) = 0$, $V''(\sigma_\infty)$ and $V'''(\sigma_\infty)$ can be written: $V''(\sigma_\infty) = -V_2\delta + O(\delta^2)$ and $V'''(\sigma_\infty) \triangleq V_3 + O(\delta)$ where V_2 and

V_3 do not depend on δ. Since $\sigma_0 - \sigma_\infty$ is $\mathrm{O}(\delta)$ we define

$$\sigma_s \triangleq \frac{\sigma - \sigma_\infty}{\delta} \tag{8}$$

Thus:

$$V(\sigma) - V(\sigma_\infty) = \left(-\frac{1}{2} V_2 {\sigma_s}^2 + \frac{1}{6} V_3 {\sigma_s}^3\right) \delta^3 \tag{9}$$

Using $V(\sigma_0) = V(\sigma_\infty)$ and defining $\bar{\tau} \triangleq \tau \cdot \sqrt{\delta}/\tau_{syn}$, Eqs. (33,9) imply:

$$\frac{\mathrm{d}\sigma_s}{\mathrm{d}\bar{\tau}} = -\sqrt{V_2 {\sigma_s}^2 - \frac{1}{3} V_3 {\sigma_s}^3}$$

Integration of this equation with the constraint that the derivative

$$\left.\frac{\mathrm{d}\sigma_s}{\mathrm{d}\bar{\tau}}\right|_{\bar{\tau}=0} = 0 \tag{10}$$

yields:

$$\sigma_s(\bar{\tau}) = \frac{3V_2}{V_3} \left[\cosh\left(\frac{\sqrt{V_2}\bar{\tau}}{2}\right)\right]^{-2} \tag{11}$$

In particular, at chaos onset the amplitude of the fluctuations in the net synaptic inputs vanishes linearly with δ whereas the correlation time of these fluctuations diverges as $1/\sqrt{\delta}$.

Finally, the coefficients V_2 and V_3 as well as $\mu^{(1)}$, $\sigma_0^{(1)}$ and $\sigma_\infty^{(1)}$ are obtained using Eq. (7) combined with $V(\sigma_0) = V(\sigma_\infty)$. Hence:

$$V''(\sigma_\infty) + \frac{1}{3} V'''(\sigma_\infty)(\sigma_0^{(1)} - \sigma_\infty^{(1)})\delta = o(\delta)$$

A tedious but straightforward calculation shows that the coefficients $\mu^{(1)}$, $\sigma_0^{(1)}$ and $\sigma_\infty^{(1)}$ are given by $\left(\mu^{(1)}, \sigma_0^{(1)}, \sigma_\infty^{(1)}\right)^T = -\frac{2}{J_c}\mathbf{W}^{-1} \cdot (1, \sigma_c, I_0/2)^T$ where

$$\mathbf{W} = \begin{bmatrix} 2{J_c}^2 R_{12} & {J_c}^2 Q \left[\frac{1}{\sqrt{\sigma_c}} + \frac{4}{3} - {J_c}^2 R_{13}\right] & -{J_c}^2 Q \left[\frac{1}{\sqrt{\sigma_c}} + \frac{2}{3} - 2{J_c}^2 R_{13}\right] \\ 2{J_c}^2 R_{01} & -{J_c}^2 R_{02} \frac{1}{\sqrt{\sigma_c}} & \left(\frac{1}{\sqrt{\sigma_c}}\left[1 + 2{J_c}^2 R_{02}\right] - 1\right) \\ J_c R_1 & \frac{1}{2} J_c R_2 & 0 \end{bmatrix}$$

and

$$R_m \triangleq \int_{-\infty}^{\infty} g^{(m)}\left(\mu_c + \sqrt{\sigma_c} z\right) \mathrm{D}z$$

$$R_{mn} \triangleq \int_{-\infty}^{\infty} g^{(m)}\left(\mu_c + \sqrt{\sigma_c} z\right) g^{(n)}\left(\mu_c + \sqrt{\sigma_c} z\right) \mathrm{D}z$$

$$Q \triangleq \int_{-\infty}^{\infty} \left[g''\left(\mu_c + \sqrt{\sigma_c} z\right)\right]^2 \mathrm{D}z$$

One then gets:

$$V_2 = {J_c}^2 \left(\frac{\sigma_0^{(1)} - \sigma_\infty^{(1)}}{\sqrt{\sigma_c}} Q + 2\mu^{(1)} R_{12} + \left(2\sigma_\infty^{(1)} - \sigma_0^{(1)}\right)\left(Q + R_{13}\right) \right) + \frac{2}{J_c} \quad (12)$$

$$V_3 = {J_c}^2 Q \quad (13)$$

Example: Sigmoid transfer function

For $g(x) \triangleq \phi(x) = \frac{1}{2}\left[1 + \mathrm{erf}\left(\frac{x}{\sqrt{2}}\right)\right]$ and $G(x) = \Phi(x) \triangleq \frac{x}{2}\left[1 + \mathrm{erf}\left(\frac{x}{\sqrt{2}}\right)\right] + \frac{e^{-\frac{x^2}{2}}}{\sqrt{2\pi}}$, μ, σ_0 and σ_∞ satisfy:

$$\sigma_\infty = {J_0}^2 \left[\phi\left(\frac{\mu}{\sqrt{1+\sigma_0}}\right) - 2T\left(\frac{\mu}{\sqrt{1+\sigma_0}}, \sqrt{\frac{1+\sigma_0-\sigma_\infty}{1+\sigma_0+\sigma_\infty}}\right) \right] \quad (14)$$

$$\frac{{\sigma_0}^2 - {\sigma_\infty}^2}{2} = \quad (15)$$

$$= {J_0}^2 \int_{-\infty}^{\infty} \left(\left[\Phi(\mu + \sqrt{\sigma_0} z)\right]^2 - (1 + \sigma_0 - \sigma_\infty)\left[\Phi\left(\frac{\mu + \sqrt{\sigma_0} z}{\sqrt{1+\sigma_0-\sigma_\infty}}\right)\right]^2 \right) \mathrm{D}z$$

and

$$I_0 - \frac{\mu}{\sqrt{K}} = J_0 \cdot \phi\left(\frac{\mu}{\sqrt{1+\sigma_0}}\right) \quad (16)$$

where $T(h,a) = \frac{e^{-\frac{h^2}{2}}}{\sqrt{2\pi}} \int_0^a \frac{1}{1+x^2} \frac{e^{-\frac{h^2 x^2}{2}}}{\sqrt{2\pi}} \mathrm{d}x$.

At chaos onset, $\sigma_0, \sigma_\infty \to \sigma_c$ and:

$$\frac{1}{2}\left(\frac{1}{{a_c}^2} - 1\right) = {J_c}^2\left(\phi(h_c)\right) - 2T(h_c, a_c) \tag{17}$$

$$1 = \frac{{J_c}^2}{2\pi} a_c \cdot e^{-\frac{1}{2}{h_c}^2\left(1+{a_c}^2\right)} \tag{18}$$

$$I_0 - \frac{\mu_c}{\sqrt{K}} = J_c \cdot \phi\left(h_c\right) \tag{19}$$

where $h_c = \mu_c/\sqrt{1+\sigma_c}$ and $a_c = 1/\sqrt{1+2\sigma_c}$.

The bifurcation diagram in Fig. 4 in the main text was obtained by numerically solving Eqs. (14)-(16) and Eqs. (17)-(19) for $I_0 = 1$. The perturbative expansion of these equations in the limit $J_0 \to J_c^+ \approx 4.995$ yields Eqs. (4) with:

$$\sigma_0^{(1)} = 2\frac{1+{a_c}^2}{1-{a_c}^2}\left[\frac{\sigma_c}{J_c} - I_0\phi\left(h_c \cdot a_c\right)\right]$$

$$\mu^{(1)} = \frac{h_c a_c \sigma_0^{(1)}}{\sqrt{2\left(1+{a_c}^2\right)}} - \frac{I_0}{{J_0}^2}\frac{\sqrt{\pi}}{a_c}\sqrt{1+{a_c}^2}e^{\frac{{h_c}^2}{2}}$$

$$\sigma_\infty^{(1)} = \frac{1}{2}\left[\frac{1}{{a_c}^2} + {h_c}^2(1+{a_c}^2) - 1\right]^{-1} \times$$
$$\times\left[\left(1 - \frac{2}{{a_c}^2} - 4{a_c}^2\left(1+{a_c}^2\right)\right)\sigma_0^{(1)} + 6\frac{h_c}{a_c}\sqrt{\frac{1+{a_c}^2}{2}}\mu_c - \frac{6}{J_c{a_c}^2}\right]$$

In particular, $\sigma_0 - \sigma_\infty = (\sigma_0^{(1)} - \sigma_\infty^{(1)})\delta + O(\delta^2)$ is an excellent match with the numerical solution of Eqs. (14)-(16) (see main text, Fig. 4A,inset).

The values of V_2 and V_3 are:

$$V_2 = -\frac{2}{J_c} +$$
$$+\, {a_c}^2\left[\sigma_0^{(1)} + \frac{1-{a_c}^2}{2{a_c}^2}(\sigma_0^{(1)} - \sigma_\infty^{(1)}) + \frac{h_c}{a_c}\sqrt{1+{a_c}^2}\mu^{(1)} - \frac{1}{2}(\sigma_0^{(1)} + \sigma_\infty^{(1)}){h_c}^2\left(1+{a_c}^2\right)\right]$$

$$V_3 = {a_c}^2\left[\frac{1}{2}{h_c}^2\left(1+{a_c}^2\right) + \frac{1-{a_c}^2}{2{a_c}^2}\right]$$

from which one obtains the PAC of the net synaptic inputs, $\sigma(\tau)$, in the vicinity of chaos onset, using Eqs. (8,11).

Figure S2 depicts the convergence of the function $(V(\sigma) - V(\sigma_\infty))/\delta^3$ to its asymptotic form in the limit $\delta \to 0$.

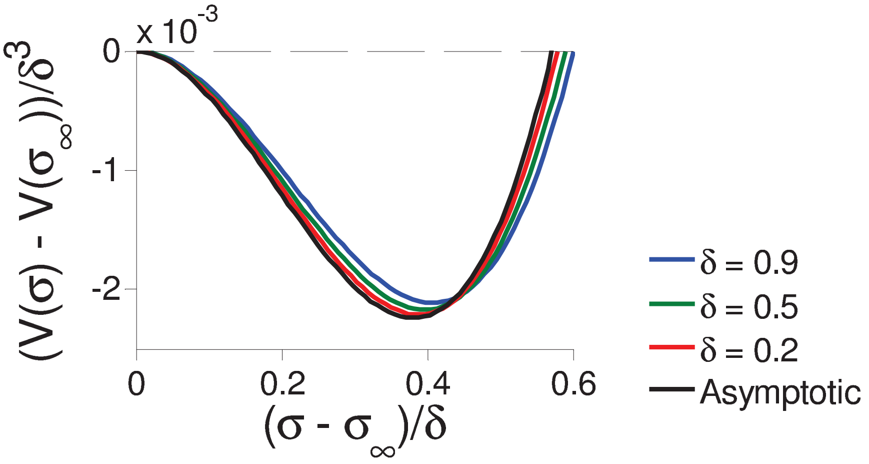

Figure S2: **The potential function in inhibitory rate models with $g(x) = \phi(x)$.** The potential was obtained for different values of $\delta = J_0 - J_c > 0$ ($J_c = 4.995$) by solving the self-consistentg DMFT equations ($I_0 = 1$). The figure shows the convergence of the potential to its asymptotic form, Eq. (9), when $\delta \to 0$.
