# Supplementary material for: Asynchronous Rate Chaos in Spiking Neuronal Circuits
Source: PLoS Comput Biol. 2015 Jul 31;11(7):e1004266. doi: 10.1371/journal.pcbi.1004266 (PMC4521798; doi:10.1371/journal.pcbi.1004266)
Supplement: S3 Text — (PDF) [file pcbi.1004266.s003.pdf]

Supporting Information for the article
"Asynchronous Rate Chaos in Spiking Neuronal Circuits"

Omri Harish and David Hansel

S3 Asynchronous rate chaos in non-leaky integrate-and-fire networks

Here we consider networks of non-leaky integrate-and-fire (NLIF) neurons. The models are the same as for LIF networks but for the dynamics of the single neurons which do not include the leak term, $-V_i^\alpha$ (see *Materials and Methods*). NLIF neurons have a threshold-linear transfer function. Figure S3 compares the PACs of NLIF spiking networks to the PACs of the corresponding rate models, i.e. with a threshold-linear transfer function. Figure S3A is for one inhibitory population whereas Fig. S3B,C are for a two-population EI network. In Fig. S3B and Fig. S3C the parameters correspond to the II and the EIE mechanism, respectively. In all cases the PACs in the spiking and in the rate model are in excellent agreement.

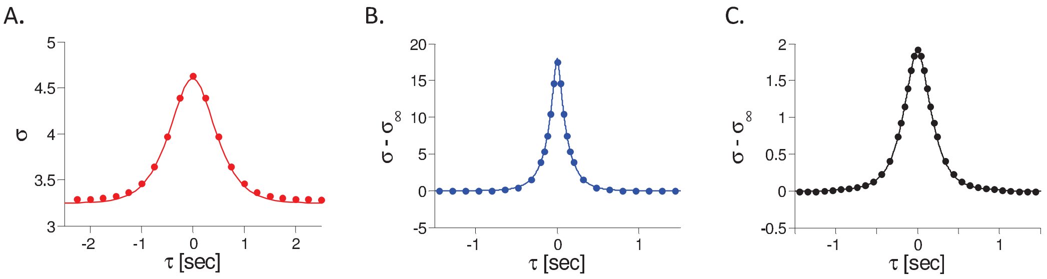

Figure S3: **PACs in non-leaky integrate-and-fire networks.** Results are from numerical simulations. Solid line: Spiking networks. Dots: Corresponding rate models. A: PACs of the net inputs to neurons in the one-population network. Parameters are $J_0 = 2$, $I_0 = 1$, $N = 256000$, $K = 1200$, $\tau_{syn} = 100$ ms. B-C: PACs of the net inputs to excitatory neurons in the two-population network. Parameters in B and C are as for the blue and black lines in Fig. S8A and B, respectively.
