# Supplementary material for: Asynchronous Rate Chaos in Spiking Neuronal Circuits
Source: PLoS Comput Biol. 2015 Jul 31;11(7):e1004266. doi: 10.1371/journal.pcbi.1004266 (PMC4521798; doi:10.1371/journal.pcbi.1004266)
Supplement: S4 Text — (PDF) [file pcbi.1004266.s004.pdf]

Supporting Information for the article
"Asynchronous Rate Chaos in Spiking Neuronal Circuits"

Omri Harish and David Hansel

S4 Onset of chaos in inhibitory rate models with threshold-power-law transfer functions

We consider networks of inhibitory neurons with transfer functions: $g(x) = x^\gamma \cdot H(x)$ where $H(x)$ is the Heaviside function ($H(x) = 1$ if $x > 0$, $H(x) = 0$ otherwise). Note that if $\gamma < 2$ this function is differentiable once but not twice, at $x = 0$. For this transfer function, Eqs. (1)-(3) read in the large K limit:

$$\sigma_c = {J_c}^2 \int_{-\mu_c/\sqrt{\sigma_c}}^{\infty} (\mu_c + \sqrt{\sigma_c} z)^{2\gamma} \mathrm{D}z \tag{20}$$

$$1 = {J_c}^2 \cdot \gamma^2 \int_{-\mu_c/\sqrt{\sigma_c}}^{\infty} (\mu_c + \sqrt{\sigma_c} z)^{2(\gamma-1)} \mathrm{D}z \tag{21}$$

$$I_0 - \frac{\mu_c}{\sqrt{K}} = J_c \int_{-\mu_c/\sqrt{\sigma_c}}^{\infty} (\mu_c + \sqrt{\sigma_c} z)^{\gamma} \, \mathrm{D}z \tag{22}$$

The integrals in the right hand sides are all finite provided that $\gamma > 1/2$. Defining

$$f_\gamma(x) \triangleq \int_{-x}^{\infty} (x+z)^\gamma \mathrm{D}z \tag{23}$$

one gets from Eqs. (20)-(21) that $x_c \triangleq \mu_c/\sqrt{\sigma_c}$ is the solution of:

$$f_{2\gamma}(x_c) - \gamma^2 f_{2(\gamma-1)}(x_c) = 0 \tag{24}$$

or equivalently:

$$(1-\gamma)^2 f_{2\gamma}(x_c) - \gamma^2 x_c f_{2\gamma-1}(x_c) = 0 \tag{25}$$

In particular, x_c does not depend on I_0.

In the limit $K \to \infty$, Eqs. (20)-(22) also imply:

$$\sigma_c = \frac{f_{2\gamma}(x_c)}{[f_\gamma(x_c)]^2} {I_0}^2 \tag{26}$$

$$J_c = \frac{[f_\gamma(x_c)]^{\gamma-1}}{[f_{2\gamma}(x_c)]^{\frac{\gamma}{2}}} {I_0}^{1-\gamma} \tag{27}$$

Therefore, once the solution of Eq. (25) is known, μ_c, J_c and σ_c are easy to obtain.

Fig. 5B in the main text plots J_c vs. γ for $I_0 = 1$. In the limit $\gamma \to (1/2)^+$, $J_c \to 0$ very sharply. In fact, in this limit x_c diverges. Specifically, writing:

$$f_\gamma(x) = x^\gamma \int_{-x}^{\infty} \left(1 + \frac{z}{x}\right)^\gamma \mathrm{D}z$$

and expanding the integral for large x one gets:

$$f_\gamma(x) \sim x^\gamma \left(1 + (\gamma - 1)\frac{e^{-\frac{x^2}{2}}}{x\sqrt{2\pi}}\right)$$

Therefore Eq. (25) reads:

$$\left(\gamma^2 - (2\gamma - 1)^2\right) \cdot \frac{e^{-\frac{{x_c}^2}{2}}}{x_c\sqrt{2\pi}} = 2\gamma - 1$$

For $\gamma = \frac{1}{2} + \epsilon$ with $0 < \epsilon \ll 1$ one has:

$$\frac{e^{-\frac{{x_c}^2}{2}}}{x_c\sqrt{2\pi}} = 8\epsilon$$

Thus $x_c \sim \sqrt{-2\log\epsilon}$, and $J_c \sim (-2\log\epsilon)^{-1/4}$.

Example: Threshold-linear transfer function

For $\gamma = 1$, $g(x) = xH(x)$ and $G(x) = \frac{x^2}{2}H(x)$. It is useful to define the functions $F_n(a,b) = \int\limits_{-a/b}^{\infty} (a+bz)^n \,\mathrm{D}z$. In particular:

$$
\begin{aligned}
F_1(a,b) &= \frac{a}{2}\left[1+\mathrm{erf}\left(\frac{a}{\sqrt{2}b}\right)\right] + \frac{b}{\sqrt{2\pi}}e^{-\frac{a^2}{2b^2}} && (28)\\
F_2(a,b) &= \frac{a^2+b^2}{2}\left[1+\mathrm{erf}\left(\frac{a}{\sqrt{2}b}\right)\right] + \frac{ab}{\sqrt{2\pi}}e^{-\frac{a^2}{2b^2}}\\
F_4(a,b) &= \frac{a^4+3b^4+6a^2b^2}{2}\left[1+\mathrm{erf}\left(\frac{a}{\sqrt{2}b}\right)\right] + \frac{a^3b+5ab^3}{\sqrt{2\pi}}e^{-\frac{a^2}{2b^2}}
\end{aligned}
$$

Equations (37), (38) and (25) then read in the large K limit:

$$\sigma_\infty = {J_0}^2 \int\limits_{-\infty}^{\infty} \left[F_1\left(\mu + \sqrt{\sigma_\infty}z, \sqrt{\sigma_0 - \sigma_\infty}\right)\right]^2 \mathrm{D}z \tag{29}$$

$$\frac{{\sigma_0}^2 - {\sigma_\infty}^2}{2} = \frac{{J_0}^2}{4}\left[F_4(\mu, \sqrt{\sigma_0}) - \int\limits_{-\infty}^{\infty} \left[F_2\left(\mu + \sqrt{\sigma_\infty}z, \sqrt{\sigma_0 - \sigma_\infty}\right)\right]^2 \mathrm{D}z\right] \tag{30}$$

$$I_0 = J_0 F_1(\mu, \sqrt{\sigma_0}) \tag{31}$$

Equations (1)-(3) yield:

$$
\begin{aligned}
\sigma_c &= {J_c}^2\left[\frac{{\mu_c}^2+\sigma_c}{2}\left(1+\mathrm{erf}\left(\frac{\mu_c}{\sqrt{2\sigma_c}}\right)\right) + \frac{\mu_c\sqrt{\sigma_c}}{\sqrt{2\pi}}e^{-\frac{{\mu_c}^2}{2\sigma_c}}\right] && (32)\\
1 &= \frac{{J_c}^2}{2}\left[1+\mathrm{erf}\left(\frac{\mu_c}{\sqrt{2\sigma_c}}\right)\right] && (33)\\
I_0 - \frac{\mu_c}{\sqrt{K}} &= J_c\left[\frac{\mu_c}{2}\left(1+\mathrm{erf}\left(\frac{\mu_c}{\sqrt{2\sigma_c}}\right)\right) + \frac{\sqrt{\sigma_c}}{\sqrt{2\pi}}e^{-\frac{{\mu_c}^2}{2\sigma_c}}\right] && (34)
\end{aligned}
$$

It is easy to see that the solution is: $\mu_c = 0, J_c = \sqrt{2}, \sigma_c = {I_0}^2\pi$. Expanding Eqs. (29)-(31) near J_c, one finds: $\mu = -\frac{\pi}{2}\delta + O(\delta^2)$ and $\sigma_0 - \sigma_\infty \propto \delta^2$. This critical behavior is different from the one we found for a sigmoid transfer function where $\sigma_0 - \sigma_\infty \propto \delta$. Therefore we will write $\sigma_0 - \sigma_\infty = a\delta^2 + O(\delta^3)$ and more generally $\sigma(\tau) - \sigma_\infty = \sigma_s(\tau)\delta^2 + O(\delta^3)$.

The second order derivative of the potential

$$V''(\sigma_\infty) = -1 + {J_0}^2\left[\phi\left(\frac{\mu}{\sqrt{\sigma_0}}\right) - 2T\left(\frac{\mu}{\sqrt{\sigma_0}}, \sqrt{\frac{\sigma_0 - \sigma_\infty}{\sigma_0 + \sigma_\infty}}\right)\right]$$

(where $T(h,a) = \frac{e^{-\frac{h^2}{2}}}{\sqrt{2\pi}} \int_0^a \frac{1}{1+x^2} \frac{e^{-\frac{h^2 x^2}{2}}}{\sqrt{2\pi}} \mathrm{d}x$) vanishes at the onset of chaos like $V_2 \delta$ with:

$$V_2 = \frac{1}{\sqrt{2}} - \frac{\sqrt{2a}}{\pi\sqrt{\pi}}$$

but all the derivatives of order $n > 2$, $V^{(n)}(\sigma_\infty)$, diverge when $\sigma_0 \to \sigma_\infty$ since:

$$V^{(n)}(\sigma_\infty) \sim (1 \cdot 3 \cdot 5 \cdot \ldots \cdot (2n-7)) \frac{{J_0}^2}{2\pi} {\sigma_\infty}^{n-3} \left({\sigma_0}^2 - {\sigma_\infty}^2\right)^{\frac{5}{2}-n} \mathrm{e}^{-\frac{\mu^2}{\sigma_0 + \sigma_\infty}}$$

Hence, to compute the shape of the potential in the limit $J_0 \to J_c^+$, we need to consider an infinite number of terms in the expansion of $V(\sigma)$ near σ_∞ and for each term keep the most diverging contribution. This yields:

$$V(\sigma) - V(\sigma_\infty) = \delta^5 \bar{V}(\sigma_s)$$

where:

$$\bar{V}(\sigma_s) = \frac{V_2}{2}{\sigma_s}^2 + \frac{V_3}{6}{\sigma_s}^3 + B \cdot \sum_{n=4}^{\infty} \frac{(2n-7)!}{2^{n-4}(n-4)!} \left(\frac{\sigma_s}{2a}\right)^n \tag{35}$$

with for $n > 2$:

$$\begin{aligned} V_n &= \frac{1}{\pi} 4\sqrt{2} {J_c}^2 {\sigma_c}^{-\frac{1}{2}} a^{\frac{5}{2}} \cdot \frac{(2n-7)!}{2^{n-4}(n-4)!} \cdot \frac{1}{2^n a^n} \\ B &= \pi^{-\frac{3}{2}} 4\sqrt{2} a^{\frac{5}{2}} \end{aligned}$$

In particular:

$$V_3 = \frac{1}{\pi\sqrt{2\pi a}}$$

Resumming the series in Eq. (35) we get:

$$\bar{V}(\sigma_s) = \frac{V_2}{2}{\sigma_s}^2 + \frac{V_3}{6}{\sigma_s}^3 - 2Bp\left(\frac{\sigma_s}{2a}\right) \tag{36}$$

with:

$$p(x) = \frac{1}{15}\left(1 - (1-2x)^{\frac{5}{2}}\right) - \frac{x}{3} + \frac{x^2}{2} - \frac{x^3}{6}$$

Hence, at chaos onset, the differential equation obeyed by $\sigma(\tau)$ reads:

$$\tau_{syn} \frac{\mathrm{d}\sigma_s}{\mathrm{d}\bar{\tau}} = -\sqrt{-V_2{\sigma_s}^2 - \frac{V_3}{3}{\sigma_s}^3 - 2Bp\left(\frac{\sigma_s}{2a}\right)} \tag{37}$$

where $\bar{\tau} \triangleq \tau\sqrt{\delta}$. The decorrelation time of the input fluctuations diverges proportionally to $1/\sqrt{\delta}$ as in the case of the sigmoid transfer function but the amplitude of the fluctuations vanishes proportionally to δ^2, rather than δ in the latter case.
Finally, the condition, $V(\sigma_0) = V(\sigma_\infty)$, determines the coefficient a:

$$a = \left(\frac{15}{24}\right)^2 \pi^3$$

and therefore

$$B = \frac{15}{6}\sqrt{2} \cdot \pi^6 \left(\frac{113}{288}\right)^2$$

Figure S4 depicts the convergence of the function $(V(\sigma) - V(\sigma_\infty))/\delta^5$ to its asymptotic form in the limit $\delta \to 0$. Note the difference between this case and the sigmoid TF case (section S2) in the scaling of $V(\sigma) - V(\sigma_\infty)$ and $\sigma - \sigma_\infty$ with δ.

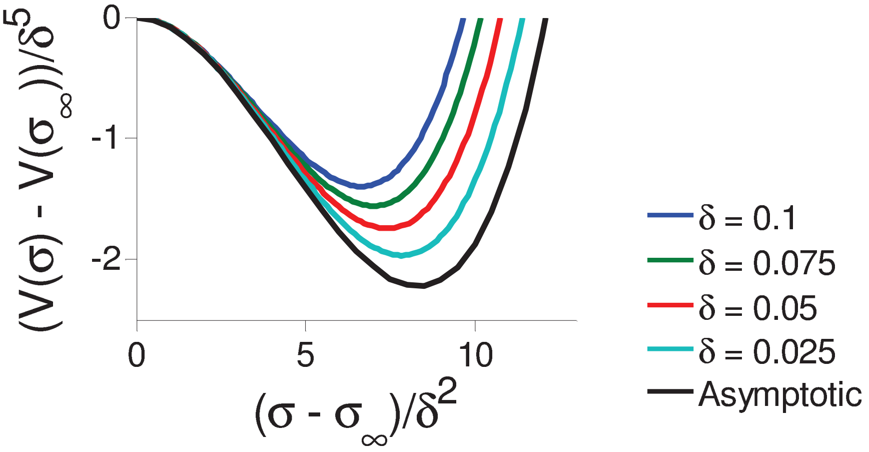

Figure S4: **The potential function in inhibitory rate models with a threshold-linear transfer function.** The potential was obtained for different values of $\delta = J_0 - J_c > 0$ ($J_c = \sqrt{2}$). The figure shows the convergence of the potential to its asymptotic form, Eq. (36), for $\delta \to 0$.
