# Supplementary material for: Asynchronous Rate Chaos in Spiking Neuronal Circuits
Source: PLoS Comput Biol. 2015 Jul 31;11(7):e1004266. doi: 10.1371/journal.pcbi.1004266 (PMC4521798; doi:10.1371/journal.pcbi.1004266)
Supplement: S5 Text — (PDF) [file pcbi.1004266.s005.pdf]

Supporting Information for the article
"Asynchronous Rate Chaos in Spiking Neuronal Circuits"

Omri Harish and David Hansel

S5 Maximum Lyapunov exponents in the inhibitory LIF rate model

Figure S5A shows histograms of the maximum Lyapunov exponent calculated for different realizations of the network and different values of J_0 (N = 40,000, K = 800, $I_0 = 1$). When J_0 is 0.9, negative Lyapunov exponents are virtually never observed. As J_0 is decreased to 0.7, the center of the distribution shifts below zero, but the distribution is wide enough to observe positive as well as negative Λ's. When J_0 is further decreased to 0.5 the distribution lies mostly in the negative part, but it has a long tail and thus positive Λ can still be observed. When $J_0 = 0.3$ the probability has a long tail toward positive Λ but the fraction of networks there becomes extremely small. These results are summarized in Fig. S5B which plots the fraction of chaotic networks vs. J_0. As a result, for $J_0 < 0.3$, simulations of the network virtually always converge to a fixed point unless the network size is extremely large.

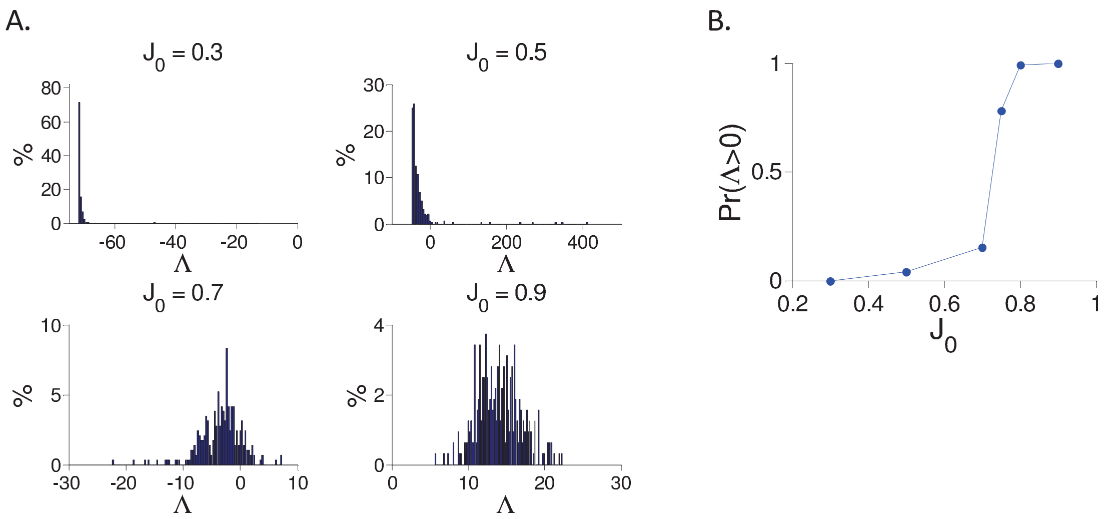

Figure S5: **Lyapunov exponent in simulations of the inhibitory LIF rate model.** Parameters: N = 40,000, K = 800, I_0=1. A: Distributions of the Lyapunov exponent, Λ, calculated over 320 realizations of the network for four values of J_0. B: The fraction of networks with $\Lambda > 0$ is plotted against J_0.
