# Supplementary material for: Asynchronous Rate Chaos in Spiking Neuronal Circuits
Source: PLoS Comput Biol. 2015 Jul 31;11(7):e1004266. doi: 10.1371/journal.pcbi.1004266 (PMC4521798; doi:10.1371/journal.pcbi.1004266)
Supplement: S6 Text — (PDF) [file pcbi.1004266.s006.pdf]

Supporting Information for the article
"Asynchronous Rate Chaos in Spiking Neuronal Circuits"

Omri Harish and David Hansel

S6 Firing statistics in the inhibitory LIF spiking network.

For all parameter values, the activity of the neurons in the inhibitory LIF spiking network is very heterogeneous across cells and highly irregular in time (Fig. 8). However, the firing patterns depend on the synaptic strength, J_0, and on the synaptic time constant, τ_{syn}. For slow and sufficiently strong synapses, neurons fire bursts of spikes. This is not the case for fast or too weak synapses as explained in the main text. Figure S6 displays additional results regarding the firing statistics of the neurons. In Fig. S6A, synapses are fast (τ_{syn} = 3 ms) and relatively strong ($J_0 = 2$). The distribution of the time-averaged firing rates of the neurons is very skewed: although the mean is about 14 Hz some neurons have a firing rate as high as 130 Hz. The distribution of the coefficient of variation (CV) of the interspike intervals peaks around CV=1: this reflects the fact that most of the neurons spike highly irregularly and without bursts. Fig. S6B shows what happens when the synapses are slow (keeping J_0 unchanged). In this case, although the distribution of neuronal firing rates does not change much, the CV distribution shifts to high values: it peaks around 5. This is because neurons now fire bursts of spikes. Reducing the synaptic strength, while keeping the synapses slow (τ_{syn} = 100 ms) affects the firing rate distribution as well as the CV distribution. Comparing panels B,C,D, shows that (i) the time-averaged firing rate increases (14.1 Hz in B, 27 Hz in C, 82Hz in D; it is inversely proportional to J_0 because the inhibitory feedback must balance the feedfoward excitation), (ii) The CV of the neurons with higher rates decreases, reflecting the fact that their patterns of activity become more tonic and more regular and (iii) the fluctuations in the synaptic inputs become very small; see also the reduction in the amplitude of the PACs in Fig. 9B in the main text. In panel D, the distribution of firing rates is almost Gaussian. There are very few neurons at low firing rates and only those neurons tend to fire bursts of spikes.

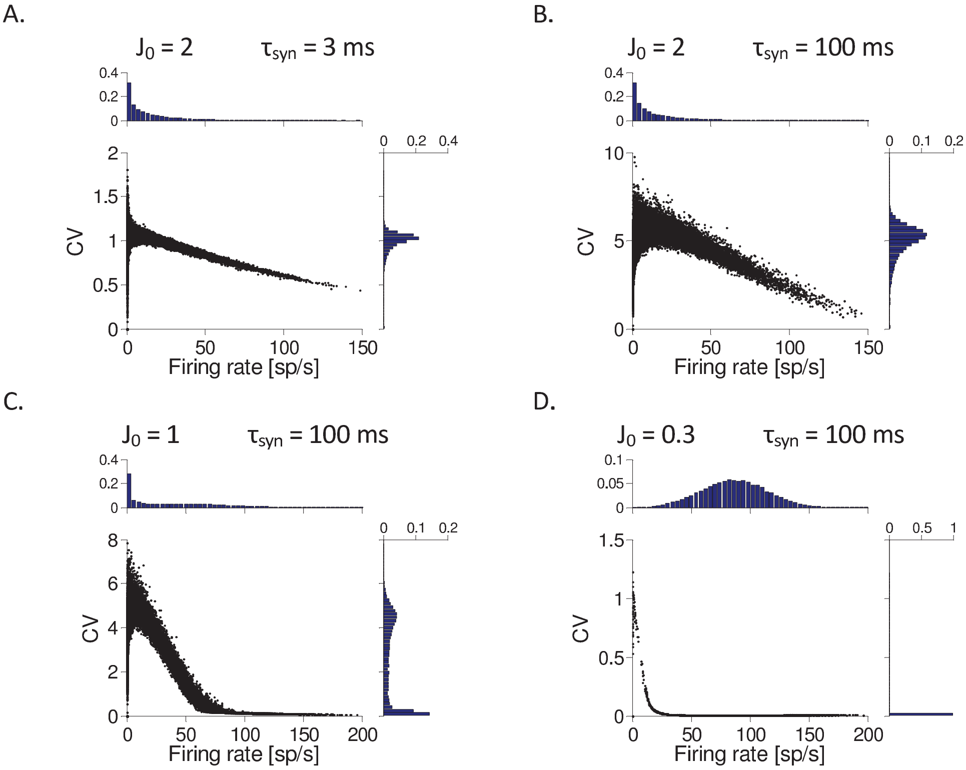

Figure S6: **Firing statistics in the LIF inhibitory spiking model.** All the results are from numerical simulations (N=40,000, K = 800; I_0 = 0.3). Central panels: Coefficients of variation, CV, of the interspike interval histograms of the neurons are plotted vs. their mean firing rates. Right panels: Distributions of the CV. Top panels: Distributions of the firing rates. A: $J_0 = 2$, $\tau_{syn} = 3$ ms. B-D: $\tau_{syn} = 100$ ms. B: $J_0 = 2$. C: $J_0 = 1$. D: $J_0 = 0.3$.
