# Supplementary material for: Asynchronous Rate Chaos in Spiking Neuronal Circuits
Source: PLoS Comput Biol. 2015 Jul 31;11(7):e1004266. doi: 10.1371/journal.pcbi.1004266 (PMC4521798; doi:10.1371/journal.pcbi.1004266)
Supplement: S7 Text — (PDF) [file pcbi.1004266.s007.pdf]

Supporting Information for the article
"Asynchronous Rate Chaos in Spiking Neuronal Circuits"

Omri Harish and David Hansel

S7 Two-population rate model with a threshold-linear transfer function with $J_0^{EE} > 0$

The results described in the main text for the two-population rate network with threshold-linear transfer function were obtained with $J_0^{EE} = 0$. We found that there are two mechanisms for the emergence of chaos in these networks. Here we extend these results to networks with $J_0^{EE} > 0$. It must be first noted that if EE synapses are strong and too fast compared to II synapses, the network dynamics can bifurcate from fixed point to oscillations (rather than to chaos). Bifurcations to oscillations are not in the topic of the present paper. Therefore we will assume that EE connections are slow enough to prevent them.

An example of the phase diagram in such a case is depicted in Fig. S7A for values of J_0^{EE} and J_0^{EI} which are comparable. As it is the case for $J_0^{EE} = 0$ chaos occurs (white region). Figure S7B-C demonstrates that the mutual inhibition (J_0^{II}) as well as the loop EIE contribute to the emergence of the chaos. To that end, we compare a reference case ($\tau_{EI} = \tau_{IE} = \tau_{II}$ = 10 ms, blue) to cases with tenfold increase in τ_{II} (green) and τ_{IE} (red). When the II connections are relatively weak (Fig. S7B) the increase in the IE synaptic time constant affects the decorrelation time more strongly than the increase in the II time constant. This indicates that here the EIE loop contributes more to the chaos generation than the II interactions (as in Fig. 15B when J_0^{EE}=0). In contrast, for large J_0^{II} the greater effect on decorrelation time is observed when τ_{II} is changed (Fig. S7C) implying that here it is the II connections that have the larger contribution to the chaotic dynamics (as in Fig. 15A when J_0^{EE}=0).

The region plotted in red in Fig S7A corresponds to the domain of existence and stability of the fixed point with strictly positive firing rates of the two populations, r_E and r_I. In this region the network state is stationary and balanced in the two populations. In the blue regions (plain and striped) the network settles in a state which is also a fixed point of the dynamics but with $r_E = 0$ and $r_I > 0$, i.e., the I population is balanced but not the E population. In the blue-striped region, this state coexists with a solution

of the balance equation with $r_E > 0, r_I > 0$. However, the latter is unstable. In the green region the network is unable to control its activity which diverges in the large K limit.

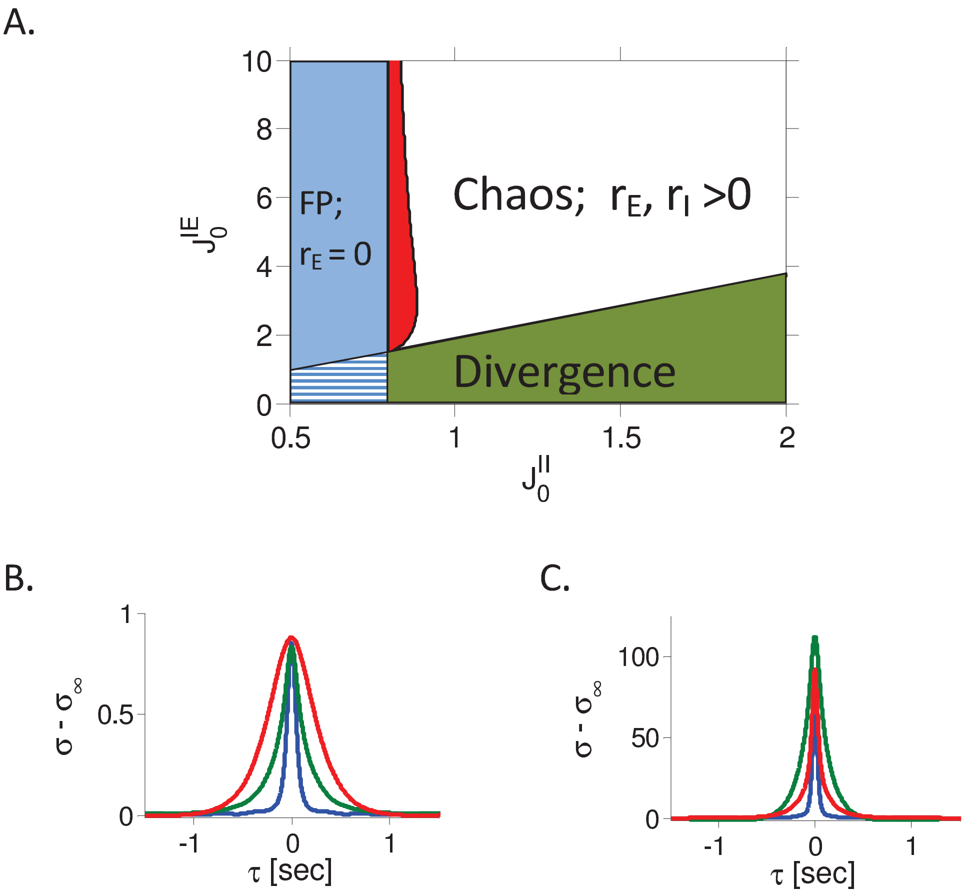

Figure S7: **Two-population rate model with a threshold-linear transfer function and EE connections.** A. The phase diagram for $J_0^{EE} = 1.5$, $J_0^{EI} = 0.8$ and $I_E = I_I = 1$ and $K \to \infty$. The vertical line at the left boundary of the red region is given by $J_0^{II} = J_0^{EI} \frac{I_I}{I_E} = 0.8$. The straight line at the lower boundary of the white region is given by: $J_0^{IE} = \frac{J_0^{EE}}{J_0^{EI}} J_0^{II} = 1.875 J_0^{II}$. Red region: The network stable state is a fixed point with $r_E > 0, r_I > 0$ in which inhibition balanced excitation in the two populations. White region: This fixed point with exists but is unstable. Inhibition balanced excitation in the two populations but the dynamics is chaotic. Blue regions: The stable network state is a fixed point with $r_E = 0, r_I > 0$. In the blue-striped region, this state coexists with a fixed point balanced state with $r_E > 0, r_I > 0$. However the latter is unstable. Green region: The activity goes to infinity in the two populations. The network is unstable (in the limit $K \to \infty$). B-C: PAC in simulations of a network with $N = 8000$, $K = 400$, $\tau_{EE} = 100$ ms, $J_{IE} = 10$, $J_{II} = 0.85$ (in B) and 4 (in C). Other parameters are as in A. Blue: Reference case, $\tau_{II} = \tau_{EI} = \tau_{IE} = 10$ ms. Green: Slow II synapses. The synaptic time constants are $\tau_{II} = 100$ ms and $\tau_{EI} = \tau_{IE} = 10$ ms. Red: Slow IE synapses. The synaptic time constants are $\tau_{IE} = 100$ ms, and $\tau_{EI} = \tau_{II} = 10$ ms. For small J_{II} (B), the decorrelation time is substantially dependent on τ_{IE} and to a lesser extent by τ_{II}. It is the opposite for large J_{II} (C).
