# Supplementary material for: Asynchronous Rate Chaos in Spiking Neuronal Circuits
Source: PLoS Comput Biol. 2015 Jul 31;11(7):e1004266. doi: 10.1371/journal.pcbi.1004266 (PMC4521798; doi:10.1371/journal.pcbi.1004266)
Supplement: S8 Text — (PDF) [file pcbi.1004266.s008.pdf]

Supporting Information for the article
"Asynchronous Rate Chaos in Spiking Neuronal Circuits"

Omri Harish and David Hansel

S8 The two mechanisms underlying asynchronous chaos in the two-population rate model with a threshold-linear transfer function.

As explained in the main text, chaos can emerge in two-population (EI) networks in two ways that differ in the nature of the interactions driving the activity fluctuations. In one of the mechanisms, II interactions are the primary drivers of the fluctuations whereas in the other mechanism, the EIE loop plays the key role. Changing the synaptic time constants affects the PACs of the net inputs into the neurons differently in the two mechanisms. This is shown for the E neurons in the main text (Fig. 15A,B) for the two-population rate model with threshold-linear transfer function. Here we show the results for the I population (Fig. S8A, II mechanism; Fig. S8 EIE mechanism).

In Fig. 15 in the main text we demonstrate the effect of enlarging the inhibitory time constant, τ_{II}, and one of the time constants in the EIE loop, namely τ_{IE}. Here, we consider the case where the EIE feedback is slow. We compare two situations: i) this feedback is slow because the EI interactions are slow (τ_{EI} large, τ_{IE} small); ii) it is slow because the IE interactions are slow (τ_{IE} large, τ_{EI} small). Figure S8C,D plot the PACs for the E (left panels in C and D) and I (right panels in C,D) neurons for large τ_{IE} (blue) and large τ_{EI} (red). In the II mechanism, (Fig. S8C), the PAC of the I population is the same whether the large time constant in the loop is τ_{IE} or τ_{EI}. This contrasts with the E population, where when the slow time constant is τ_{EI} the amplitude is smaller and the width is larger. This is because in the II mechanism, the fluctuations generated within the I population are filtered out by slow inhibitory-to-excitatory transmission. As a result, the PAC of the E population becomes wider and its amplitude smaller when τ_{EI} is increased. By contrast, in the EIE mechanism (Fig. S8D) the two synaptic time constants of the EIE loop have the same effect on the PACs, since the chaos is generated by the loop.

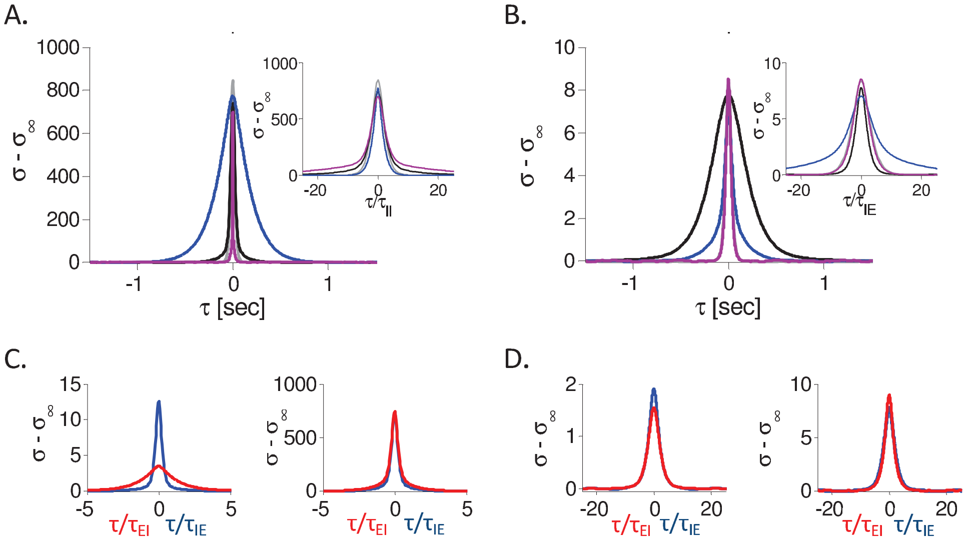

Figure S8: **The two mechanisms underlying chaos in the two-population rate model with a threshold-linear transfer functions.** A-B: PACs of the net inputs to the inhibitory neurons for different synaptic time constants: (i) $\tau_{IE} = \tau_{EI} = \tau_{II} = 10$ ms (gray); (ii) $\tau_{IE} = 100$ ms, $\tau_{EI} = \tau_{II} = 10$ ms (black); (iii) $\tau_{II} = 100$ ms, $\tau_{EI} = \tau_{IE} = 10$ ms (blue); (iv) $\tau_{II} = 1$ ms, $\tau_{EI} = \tau_{IE} = 10$ ms (purple). Insets: All PACs are plotted vs. τ/τ_{II} (in A) and τ/τ_{IE} (in B). A: II mechanism. $J_0^{II} = 6$, $J_0^{IE} = 10$. B: EIE mechanism. $J_0^{II} = 1$, $J_0^{IE} = 15$. Other parameters: $I_E = I_I = 1$, $J_0^{EE} = 0$, $J_0^{EI} = 0.8$. $N_E = N_I = 8000$, $K = 400$. The corresponding results for the PAC of the net inputs to the excitatory neurons are plotted in Fig. 15. C: PACs of the net inputs to the excitatory (left panel) and inhibitory neurons (right panel) for J_0^{EE} =0, J_0^{EI} =0.8, $J_0^{II} = 6$, $J_0^{IE} = 10.$, $I_E = I_I$ =1. Blue: τ_{IE}=100 ms, $\tau_{EI} = \tau_{II} = 10$ ms. The PACs are plotted vs. τ/τ_{IE}. Red: τ_{EI}=100 ms and τ_{IE}=τ_{II} = 10 ms. The PACs are plotted vs. τ/τ_{EI}. D: The same as in C but for $J_0^{II} = 1$, $J_0^{IE} = 15$.
