# Supplementary material for: Asynchronous Rate Chaos in Spiking Neuronal Circuits
Source: PLoS Comput Biol. 2015 Jul 31;11(7):e1004266. doi: 10.1371/journal.pcbi.1004266 (PMC4521798; doi:10.1371/journal.pcbi.1004266)
Supplement: S9 Text — (PDF) [file pcbi.1004266.s009.pdf]

Supporting Information for the article
"Asynchronous Rate Chaos in Spiking Neuronal Circuits"

Omri Harish and David Hansel

S9 The two mechanisms underlying asynchronous chaos in two-population LIF networks: Results for inhibitory neurons.

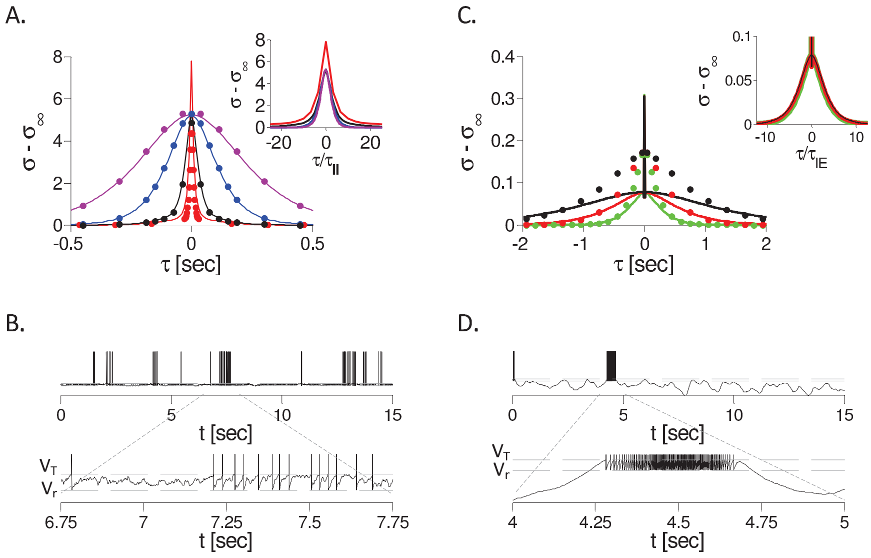

Figure S9: **The PAC for the inhibitory neurons in the mechanisms underlying chaos in two-population LIF spiking and rate networks.** Corresponding results for the excitatory populations are shown in Fig. 16 in the main text. A,B: J_0^{II}=4, τ_{IE}=100 ms. τ_{EI}=3 ms; In A the PACs of the net inputs to the *inhibitory* population are plotted for LIF spiking (solid) and rate (dots) networks for τ_{II}=3, (red), 10 (black), 40 (blue) and 100 ms (purple). Inset: All PACs (spiking network) are plotted vs. τ/τ_{II}. Panel B plots the voltage of one inhibitory neuron for parameters as for the purple line in A. C,D: J_0^{II}=1, $\tau_{EI} = \tau_{II}$=3 ms. The PAC plotted in C are for τ_{IE}=100, (green), 200 (red) and 400 ms (black). Inset: All PACs (spiking network) are plotted vs. τ/τ_{IE}. In D the voltage is plotted for one inhibitory neuron with parameters as for the green line in C. Other parameters in A-D are: N_E=N_I=16000, K=400, I_E=0.2, I_I=0.1, J_0^{EE}=0, J_0^{EI}=0.8, J_0^{IE}=3.
