# Supplementary material for: Asynchronous Rate Chaos in Spiking Neuronal Circuits
Source: PLoS Comput Biol. 2015 Jul 31;11(7):e1004266. doi: 10.1371/journal.pcbi.1004266 (PMC4521798; doi:10.1371/journal.pcbi.1004266)
Supplement: S10 Text — (PDF) [file pcbi.1004266.s010.pdf]

Supporting Information for the article
"Asynchronous Rate Chaos in Spiking Neuronal Circuits"

Omri Harish and David Hansel

S10 Two-population LIF rate and spiking models: In the II mechanism the PAC depends very mildly on τ_{IE}

The parameters of Fig. S10 correspond to the II mechanism. The PACs barely change upon decreasing τ_{IE} from 100 ms (red) to τ_{IE}=10 ms (black) in the spiking as well as in the rate model. Note that in the spiking network the PAC of the E neurons exhibits a sharp peak on a short time scale. This corresponds to fast fluctuations generated by the spiking dynamics in the I population which are conveyed by the fast EI synapses to the E neurons. This sharp peak is not present in the corresponding rate model.

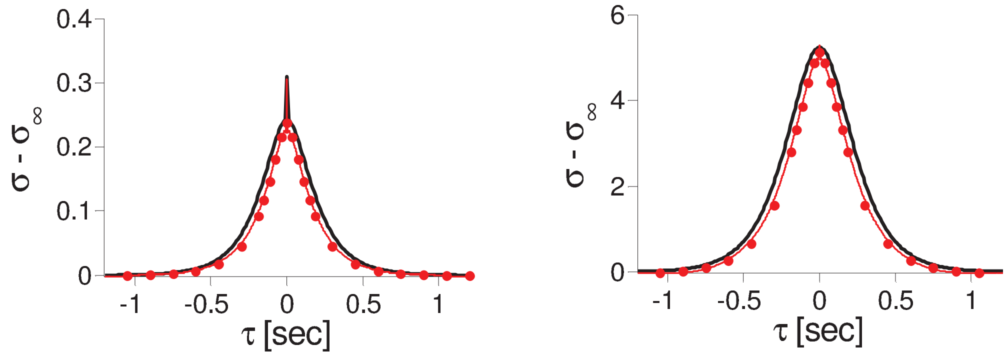

Figure S10: **The PAC depends very mildly on τ_{IE} in the II mechanism** PACs of the net inputs to excitatory (left) and inhibitory (right) neurons are plotted. All results are from numerical simulations. Parameters: $N_E = N_I = 16000$, $K = 400$, I_0^E=0.2, $I_0^I = 0.1$, J_0^{EE}=0, J_0^{EI}=0.8, J_0^{IE}=3, J_0^{II}=4, τ_{EI}=3 ms; see also Fig. 16A in the main text. Solid lines and dots are for the LIF spiking and the rate models, respectively. τ_{II}=100 ms; τ_{IE}=100 ms (red), 10 ms (black).
