# Supplementary material for: Asynchronous Rate Chaos in Spiking Neuronal Circuits
Source: PLoS Comput Biol. 2015 Jul 31;11(7):e1004266. doi: 10.1371/journal.pcbi.1004266 (PMC4521798; doi:10.1371/journal.pcbi.1004266)
Supplement: S11 Text — (PDF) [file pcbi.1004266.s011.pdf]

Supporting Information for the article
"Asynchronous Rate Chaos in Spiking Neuronal Circuits"

Omri Harish and David Hansel

S11 Two-population LIF rate and spiking models: In the EIE mechanism the slow component of the PAC depends very mildly on τ_{II}

In Fig. S11 the chaos is driven by the EIE loop. The spiking network PACs exhibit a sharp peak on a short time scale and a broad one on a long time scale. As τ_{II} increases, the fast component disappears and the amplitude of the slow component increases. By contrast, the width of the slow component depends only weakly on τ_{II} (Fig. S11, insets). This is because the chaos is driven by the EIE loop. In the corresponding rate model the PAC changes also only mildly with τ_{II}. Quantitative agreement between the spiking and the rate models is very good for τ_{II} >10 ms. The discrepancy when τ_{II} ≤10 ms (green,black) stems from the fact that the transfer function in the simulated rate model is that of the noiseless LIF neuron whereas in the spiking network, the transfer function of the neurons is effectively modulated by the rapidly fluctuating noise induced by II synapses when they are fast.

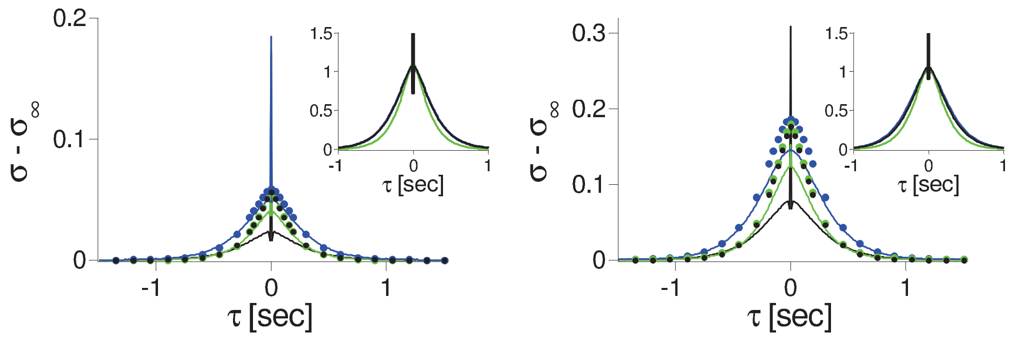

Figure S11: **In the EIE mechanism the slow component of the PAC depends very mildly on** τ_{II} PACs of the net inputs to excitatory (left) and inhibitory (right) neurons are plotted. All results are from numerical simulations. Parameters are: $N_E = N_I = 16000$, $K = 400$, I_0^E=0.2, $I_0^I = 0.1$, J_0^{EE}=0, J_0^{EI}=0.8, J_0^{IE}=3, J_0^{II}=1, τ_{IE}=100 ms, τ_{EI}=3 ms; see also Fig. 16B in the main text. Solid lines and dots are for the spiking and the rate models. τ_{II} =3 (black), 10 (green), 100 (blue). Insets: All PACs are normalized to the amplitude of their slow component.
