# Supplementary material for: Asynchronous Rate Chaos in Spiking Neuronal Circuits
Source: PLoS Comput Biol. 2015 Jul 31;11(7):e1004266. doi: 10.1371/journal.pcbi.1004266 (PMC4521798; doi:10.1371/journal.pcbi.1004266)
Supplement: S12 Text — (PDF) [file pcbi.1004266.s012.pdf]

Supporting Information for the article
"Asynchronous Rate Chaos in Spiking Neuronal Circuits"

Omri Harish and David Hansel

S12 Two-population integrate-and-fire network with recurrent EE excitation, AMPA and NMDA synapses and fast inhibition.

The results reported here extend those depicted in Fig. 16 to the case where there is substantial recurrent excitation in the E population whereas EE as well as IE excitation have two components, one fast ("AMPA") and one slow ("NMDA"). The parameters used in the simulations depicted in Fig. S12 are:

- $N_E = N_I$=16000; K=400.
- AMPA: J_0^{EE}=0.5; J_0^{IE}=3; $\tau_{EE} = \tau_{IE}$=3ms
- NMDA: J_0^{EE}=0.5; J_0^{IE}=3; $\tau_{EE} = \tau_{IE}$=100ms
- Inhibition: J_0^{EI}=1.6; J_0^{II}=2; $\tau_{IE} = \tau_{II}$=3ms
- Background input: $I_E = 0.4$; $I_I = 0.2$.

Single neuron parameters are as in Fig. 16 (main text). The slow components in the PAC of the net inputs to the neurons and in the autocorrelations of the spike trains stem from the slow dynamics of the EIE feedback loop.

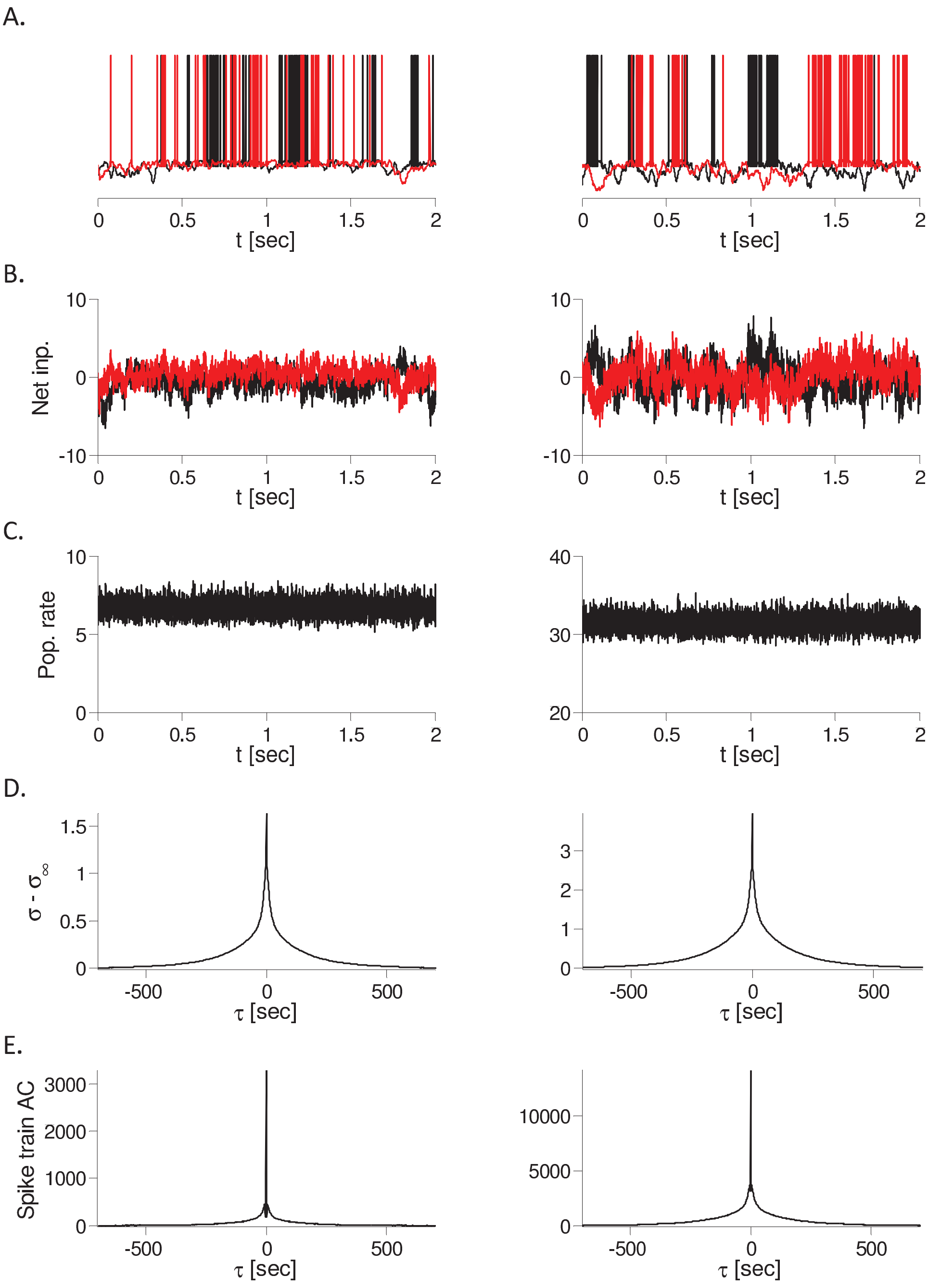

Figure S12: **Two-population integrate-and-fire network with AMPA and NMDA synapses.** Left: Excitatory neurons. Right: Inhibitory neurons. A: Voltage traces of two neurons. B: Net input to the neurons in A. C: Population-averaged firing rate in 0.1 ms time bins. D: PAC of the net input to the neurons. E: Population-averaged autocorrelation of the neuronal spike trains smoothed with an exponential filter with time constant 1 ms. (baseline subtracted).
